# Land usage attributed to corn ethanol production in the United States: sensitivity to technological advances in corn grain yield, ethanol conversion, and co-product utilization

**DOI:** 10.1186/1754-6834-7-61

**Published:** 2014-04-12

**Authors:** Rita H Mumm, Peter D Goldsmith, Kent D Rausch, Hans H Stein

**Affiliations:** 1 Department of Crop Sciences, University of Illinois at Urbana-Champaign, Urbana, IL 61801, USA; 2 Department of Agricultural and Consumer Economics, University of Illinois at Urbana-Champaign, Urbana, IL 61801, USA; 3 Department of Agricultural and Biological Engineering, University of Illinois at Urbana-Champaign, Urbana, IL 61801, USA; 4 Department of Animal Sciences, University of Illinois at Urbana-Champaign, Urbana, IL 61801, USA

**Keywords:** Agricultural biotechnology, Corn ethanol, Corn ethanol co-products, Corn gluten feed, Corn gluten meal, Corn grain production, DDGS, Distillers dried grains with solubles, Livestock feeding, Technological change

## Abstract

**Background:**

Although the system for producing yellow corn grain is well established in the US, its role among other biofeedstock alternatives to petroleum-based energy sources has to be balanced with its predominant purpose for food and feed as well as economics, land use, and environmental stewardship. We model land usage attributed to corn ethanol production in the US to evaluate the effects of anticipated technological change in corn grain production, ethanol processing, and livestock feeding through a multi-disciplinary approach. Seven scenarios are evaluated: four considering the impact of technological advances on corn grain production, two focused on improved efficiencies in ethanol processing, and one reflecting greater use of ethanol co-products (that is, distillers dried grains with solubles) in diets for dairy cattle, pigs, and poultry. For each scenario, land area attributed to corn ethanol production is estimated for three time horizons: 2011 (current), the time period at which the 15 billion gallon cap for corn ethanol as per the Renewable Fuel Standard is achieved, and 2026 (15 years out).

**Results:**

Although 40.5% of corn grain was channeled to ethanol processing in 2011, only 25% of US corn acreage was attributable to ethanol when accounting for feed co-product utilization. By 2026, land area attributed to corn ethanol production is reduced to 11% to 19% depending on the corn grain yield level associated with the four corn production scenarios, considering oil replacement associated with the soybean meal substituted in livestock diets with distillers dried grains with solubles. Efficiencies in ethanol processing, although producing more ethanol per bushel of processed corn, result in less co-products and therefore less offset of corn acreage. Shifting the use of distillers dried grains with solubles in feed to dairy cattle, pigs, and poultry substantially reduces land area attributed to corn ethanol production. However, because distillers dried grains with solubles substitutes at a higher rate for soybean meal, oil replacement requirements intensify and positively feedback to elevate estimates of land usage.

**Conclusions:**

Accounting for anticipated technological changes in the corn ethanol system is important for understanding the associated land base ascribed, and may aid in calibrating parameters for land use models in biofuel life-cycle analyses.

## Background

As alternatives to petroleum-based energy sources are sought in the US, great attention has been given to renewable fuel sources from agriculturally produced biofeedstocks, for example, miscanthus, switchgrass, sugar cane, rapidly growing tree species, and corn. Renewable fuel sources not only reduce US dependence on foreign sources for energy, but support environmental stewardship through reduction of greenhouse gas production and promote rural development.

**Figure 1:**
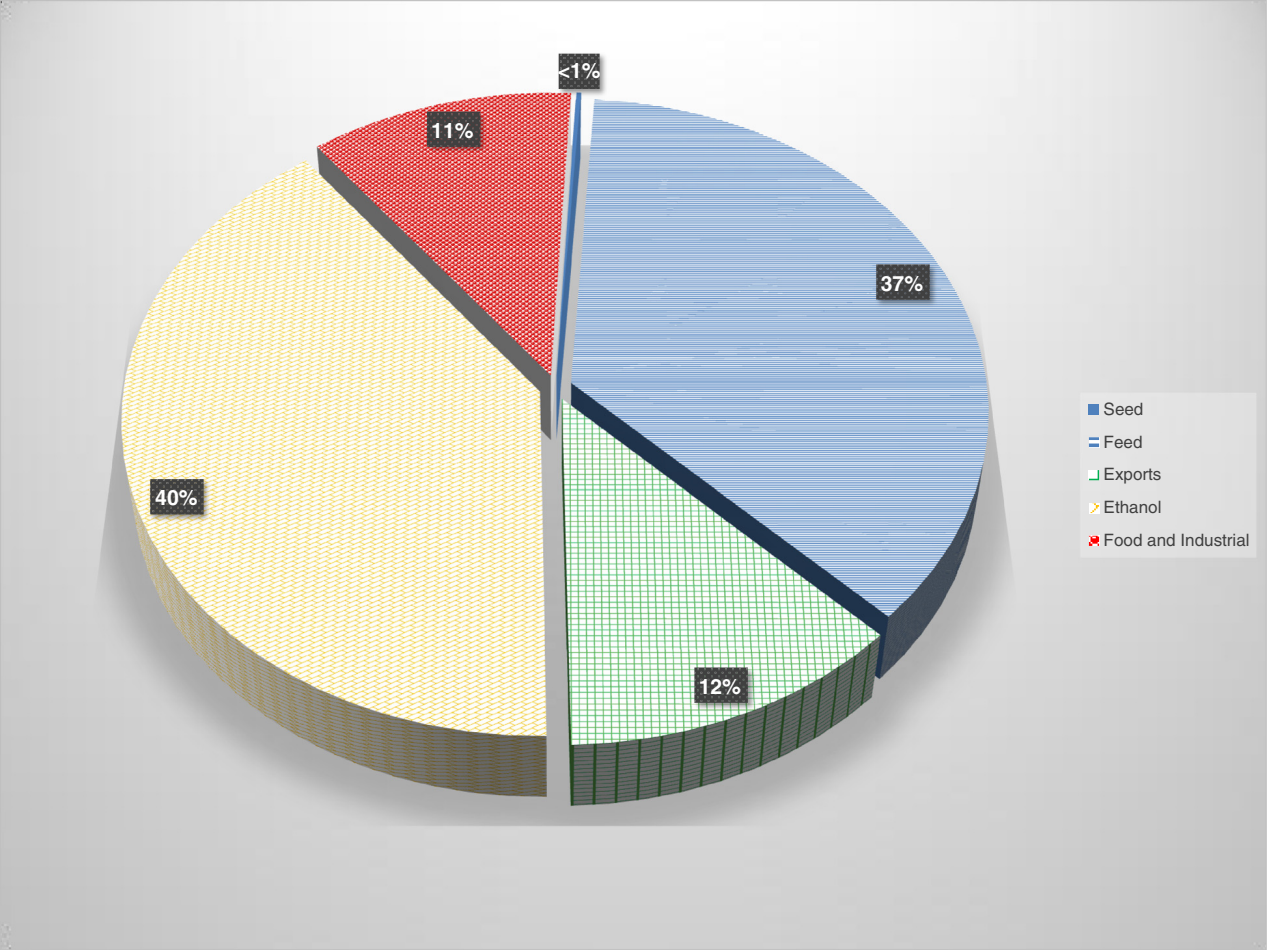
**Disposition (%) among major uses of No. 2 yellow corn harvested in the US in 2011 **[1]**.**

The system for producing and processing of corn grain (that is, US No. 2 yellow corn) is well established in the US and corn grain has been used as a source of biofuel for decades. According to the US Department of Agriculture (USDA), of the 12.360 billion bushels of corn grain harvested in 2011, more than 40% (5.007 billion bushels) was processed to produce ethanol while 37% went to livestock feed, 11% to food and industrial uses, and 12% was exported (Figure 1) [1]. The use of corn grain among other biofeedstocks has to be balanced with its longtime predominant purpose for food and feed, and other issues such as economic impacts affecting global food prices, land use, and environmental effects. The Energy Independence and Security Act adopted in 2007 established the Renewable Fuel Standard, recognizing the role of corn grain for ethanol production along with biofeedstocks for cellulosic fermentation [2]. The Renewable Fuel Standard established a limit of 15 billion gallons for the use of corn grain for ethanol. The corn ethanol system produces significant quantities of co-products, including distillers dried grains with solubles (DDGS), corn gluten feed (CGF), and corn gluten meal (CGM). These co-products substitute for corn grain and soybean meal in livestock feed, mitigating to some extent the trade-off between fuel and feed with corn grain channeled to ethanol production.

We examined three key ‘supply’ variables to quantify the long-term effects and interactions involving corn grain yield, ethanol processing, and livestock feeding to illustrate how these factors affect land area attributed to corn ethanol production. In particular, we assessed the impact of technology as it relates to changes in corn yields due to genetic and agronomic advancements, in ethanol processing due to more efficient fermentation resulting in greater ethanol output, and in changes in livestock feeding practices for beef cattle, dairy cattle, pigs, and poultry. To accomplish this, we first created a model of the US corn ethanol system featuring inputs and outputs involving production of corn grain, processing co-products, livestock feeding, and oil for biofuels.

Secondly, we developed and explored seven scenarios representing various levels of efficiency due to anticipated technological changes in corn production, ethanol processing, and livestock feeding. The seven scenarios feature: 1) corn yield estimates based on historical performance as well as publically shared information about industrial seed corn product pipelines and future product expectations which feature biotechnological advancements *i.e.* corn yield technology, 2) corn yield estimates (as in Scenario 1) minus 10% to represent a lower-bound estimate of yield with technological advancements, 3) corn yield estimates (as in Scenario 1) plus 10% to represent a upper-bound estimate of yield with technological advancements, and 4) corn yield estimates based on USDA projections which reflect mainly conventional plant breeding practices and take little account of biotechnological advancements; 5) ethanol processing advancements that feature conversion of all starch in the grain and 6) ethanol processing advancements that feature complete fiber conversion; and 7) an average livestock feeding profile associated with a 65%: 35% corn grain to soybean meal substitution ratio representing a shift toward more use of DDGS for dairy cattle, pigs, and poultry.

Thirdly, we considered three time horizons at which to estimate land use attributable to corn ethanol: 2011 (current); the time period at which the 15 billion gallon cap for corn ethanol is achieved; and 2026 (15 years out). With substituting DDGS in livestock diets, we computed land area attributable to corn ethanol accounting for the replacement of soybean oil that would otherwise be derived from DDGS-replaced soybeans, because typically that soybean oil would be directed to biofuel. We also computed land area estimates without accounting for soybean oil replacement.

Thus, we considered effects of three technology factors on the supply of corn and feed co-products within the US corn ethanol system. The analysis employed a ‘micro’ approach in that the research directly involved discipline specialists and, as a result, integrated discipline-specific insights on the behavior of the production, processing, and feeding components of the corn-ethanol system and the rapid rate of scientific advancement. Furthermore, although other studies have considered the effects of these supply variables individually to some extent (for example*,* see [3-9]), our view is from a technological perspective and one that considers the interaction among these factors within a particular geography. With this approach, we aim to provide a basis to aid in calibrating parameters for land use models and inform stakeholders of the importance of technological change in biofuel life-cycle analysis.

We limited ethanol demand in the model to a maximum of 15 billion gallons, which corresponds to the maximum allowed for first generation biofuels under the 2007 Renewable Fuels Standard. The capping of demand, while reasonable over the near and medium term, also allows the analysis to solely focus on technological advances corresponding to production, processing, and livestock feeding. Capping demand allows us to limit our scenario analysis to seven but does not allow for analysis of demand or trade scenarios. Moreover, we do not address the implications of corn stover as a biofeedstock as this depends on cellulosic fermentation technology, which is presently in its infancy [10].

## Results and discussion

Scenarios 1 through 4 explore the impact of increasing corn grain yields over the 15-year period through 2026, with the rate of yield increase influenced by the implementation and farmer adoption of yield technologies. Together, these four scenarios represent the range of growth in corn grain production over the next 15 years. Replacing corn and soybean meal in livestock diets with DDGS, CGF, and CGM has the effect of reducing the land area attributed to corn ethanol production; thus, the land area attributed to corn ethanol production is less than the acreage associated with production of the 40.5% of the corn grain directed to ethanol processing (Table 1). In 2011, not accounting for soybean oil replacement, land area for corn ethanol was 13.9 million acres, 17% of the total 83.98 million total corn acres, compared to 40.5% of all corn grain directed to ethanol processing. Because 2011 yield estimates were based on actual figures, not forecasts, the land area attributed to corn ethanol was the same across all four yield scenarios.

**Table 1:** Estimated land area attributed to corn ethanol production expressed as acreage and percent of US land dedicated to corn grain production for each of seven scenarios, without and with oil replacement, for three time horizons

		**1**	**2**	**3**	**4**	**5**	**6**	**7**
		**Corn grain production**				**Ethanol processing**		**Livestock feeding**
		**Yield technology**						
	**Time horizon**	**Medium**	**Low**	**High**	**Minimal**	**Full starch**	**Complete fiber**	**65%:35% substitution ratio**
Acres without oil replacement (millions)	2011	13.9	13.9	13.9	13.9	15.0	17.6	11.9
	Ethanol ceiling	11.0 (2013)	13.0 (2015)	8.8 (2013)	11.6 (2013)	11.8 (2013)	14.0 (2013)	8.8 (2013)
	2026	3.6	5.3	2.0	8.2	4.4	7.0	1.2
Percent of US corn land without oil replacement	2011	17%	17%	17%	17%	18%	21%	14%
	Ethanol ceiling	13% (2013)	16% (2015)	10% (2013)	14% (2013)	14% (2013)	17% (2013)	10% (2013)
	2026	4%	6%	2 %	10%	5%	8%	1%
Acres with oil replacement (millions)	2011	20.9	20.9	20.9	20.9	21.5	22.4	20.5
	Ethanol ceiling	18.4 (2013)	20.4 (2015)	16.2 (2013)	19.0 (2013)	18.6 (2013)	18.8 (2013)	17.9 (2013)
	2026	11.0	12.9	9.4	15.6	11.3	11.8	10.3
Percent of US corn land with oil replacement	2011	25%	25%	25%	25%	26%	27%	24%
	Ethanol ceiling	22% (2013)	24 % (2015)	19 % (2013)	23% (2013)	22% (2013)	22% (2013)	21% (2013)
	2026	13%	15%	11%	19%	13%	14%	12%

Accounting for soybean oil replacement, land area attributed to corn ethanol production in 2011 was 20.9 million acres, 25% of the total 83.98 million corn acres, instead of 40.5% of all corn grain directed to ethanol processing (Table 1). However, the replacement of soybean oil through canola production contributes positive feedback^a^ to the system, meaning that more land area is attributed to corn ethanol than when not accounting for oil replacement.

As corn yields increase over time, greater quantities of co-products for livestock feeding are produced in the US corn ethanol system, offsetting more land area attributed to ethanol production. In Scenario 1 (medium level of yield technology), land area attributed to corn ethanol production falls to 3.6 million acres by 2026, which is only 4% of all US land area devoted to corn production without oil replacement (Table 1). However, soybean oil replacement partially offsets the effects higher corn yields have on land usage. Although oil skimming occurring at 50% of the dry grind plants at the rate of 0.24 pounds per bushel of processed corn negatively feeds back into the system to mitigate some of the oil replacement by canola, the benefit is marginal because the volumes skimmed are relatively low. Land usage attributed to corn ethanol production in 2026 (Scenario 1) is more than three times greater when accounting for soybean oil replacement (4% versus 13%). Still, the terminal result dramatically differs from the 40.5% of the grain directed to corn ethanol.

Yield growth assumptions are critical to land usage estimates. There is a difference of 8 percentage points or more than 6 million acres when comparing no/minimal yield technology (Scenario 4) with a high level of yield technology (Scenario 3) in 2026 (with or without oil replacement). Scenario 2 is the only scenario in which the 15 billion gallon ethanol ceiling is reached in 2015; for all other yield scenarios, the ceiling is reached in 2013. Meeting this ceiling occurs when national average corn yields rise to levels in the range of 155.0 to 157.1 bushels per acre (bu/A).

The array of corn yield forecasts provided by the four levels of yield technology implementation and adoption (Tables 2 and 3) is not inconsistent with other predictions of US corn yield averages, especially with technological improvements. Scenario 1 culminates in a 244.3 bu/A average whereas Scenarios 2 and 4, which are similar in depicting a negligible impact of yield technology, average 206.0 bu/A in 2026. A similar range (205 to 242 bu/A) was predicted for the US by 2030 by Miranowski *et al*., taking into account corn grain yield performance and forecasts by state [11]. Downing *et al*. predicted that corn yield growth will result in a considerably greater supply of corn grain, with a strong likelihood of a doubling in annual increase due to technology [12]. Even across the entire range of yield estimates provided by the four yield technology scenarios, corn grain yield is shown to be a key factor influencing land usage attributed to corn ethanol production.

**Table 2:** Scenario 1 corn yield forecast (bu/A) by year through 2026 and contributing technology factors with associated step changes

**Year of maximum adoption for specified technology factor**^**a**^	**Year**	**Conventional breeding**	**Advanced breeding technology**^**b**^	**Sub-total**	**Biotechnology traits**^**c**^	**Sub-total**	**Agronomic improvements**^**d**^	**Total corn yield**
	2011^e^	147.2	0	147.2	0	147.2	0	147.2
	2012^e^	123.4	0	123.4	0	123.4	0	123.4
	2013	169.9	0.25	170.2	0	170.2	0	170.2
	2014	171.7	0.50	172.2	0	172.2	0	172.2
1	2015	173.5	1.00	174.5	0	174.5	0	174.5
3	2016	175.3	2.00	177.3	0	177.3	3	180.3
	2017	177.1	3.00	180.1	0	180.1	3	183.1
2	2018	178.9	4.00	182.9	10	192.9	3	195.9
	2019	180.7	5.00	185.7	10	195.7	3	198.7
3	2020	182.5	6.00	188.5	10	198.5	6	204.5
	2021	184.3	7.00	191.3	10	201.3	6	207.3
2	2022	186.1	8.00	194.1	20	214.1	6	220.1
	2023	187.9	9.00	196.9	20	216.9	6	222.9
3	2024	189.7	10.00	199.7	20	219.7	9	228.7
2	2025	191.5	11.00	202.5	30	232.5	9	241.5
	2026	193.3	12.00	205.3	30	235.3	9	244.3

^a^Note that multiple waves are anticipated for some technology factors. ^b^Advanced breeding technology comprises genomic-based approaches to crop improvement including DNA sequencing, molecular markers, and doubled haploidy. This class of technologies does not include genetic engineering. ^c^Biotechnology traits comprises value-added characteristics manifested through genetic modification. ^d^Agronomic improvements comprise cultural production practices that relate to the way the corn crop is managed. ^e^Actual (not forecasted) US average yields provided for this year.

**Table 3:** Corn yield forecasts (bu/A) by year from 2013 through 2026 for Scenario 1 (medium yield technology), Scenario 2 (low yield technology), Scenario 3 (high yield technology), and Scenario 4 (no/minimal technology change factors)

**Year**	**Scenario 1**	**Scenario 2**	**Scenario 3**	**Scenario 4**
2011^a^	147.2	147.2	147.2	147.2
2012^a^	123.4	123.4	123.4	123.4
2013	170.2	153.1	187.2	166.0
2014	172.2	155.0	189.4	168.0
2015	174.5	157.1	192.0	170.0
2016	180.3	162.3	198.3	172.0
2017	183.1	164.8	201.4	174.0
2018	195.9	176.3	215.5	176.0
2019	198.7	178.8	218.6	178.0
2020	204.5	184.1	225.0	180.0
2021	207.3	186.6	228.0	182.0
2022	220.1	198.1	242.1	184.0
2023	222.9	200.6	245.2	186.0
2024	228.7	205.8	251.6	188.0
2025	241.5	217.4	265.7	190.0
2026	244.3	219.9	268.7	192.0

^a^All scenarios incorporate actual average yields for 2011 and 2012.

Scenarios 5 and 6 reveal the impact of increasing efficacy in ethanol processing on land usage for corn ethanol production. Two effects stand out: the direct effect is that improved ethanol processing increases the amount of ethanol per bushel of grain, which in turn reduces the amount of land needed to meet the 15 billion gallon cap. But improving ethanol output introduces a significant positive feedback force that *ceteris paribus* raises land usage. Small increases in ethanol output dramatically reduce the volume of co-products available for livestock feeding.

With Scenario 5 involving improved starch conversion efficiency, the ethanol yield increases from 2.759 to 2.829 gallons per bushel of corn. Correspondingly, the quantity of DDGS produced as a co-product in ethanol processing decreases from 17.44 to 16.38 pounds per bushel of processed corn (Tables 4 and 5). Scenario 5 presents an interesting trade-off in keeping with findings by Mueller and Kwik [13]: a net 2.5% increase in ethanol yield per bushel of corn processed for a net 6.1% decrease in the quantity of DDGS produced per bushel. The decrease in feed co-products results in less corn and soybean being substituted, which in turn raises land area attributed to corn ethanol production. In 2011, without oil replacement, land usage is 15.0 million acres, or 18% of the total 83.98 million acres used for US corn grain production, as compared to 17% with Scenario 1 involving baseline ethanol processing efficiencies (Table 1). Including oil replacement raises land area attributed to corn ethanol with full starch conversion to 21.5 million acres, 26% of the US corn grain-producing land. Therefore, increasing ethanol output per bushel of corn by extracting more from the starch component of the grain increases land area attributed to corn ethanol in Scenario 5 compared to Scenario 1, which is a surprising result.

**Table 4:** **Composition of feed co-products**^**a**^**from ethanol processing scenarios, assuming 86% dry grind with 50% skimming oil and 14% wet milling**

	**Distillers dried grains with solubles**		**Corn gluten feed**		**Corn gluten meal**	
	**Protein (%)**	**Fat (%)**	**Protein (%)**	**Fat (%)**	**Protein (%)**	**Fat (%)**
Baseline^b^	27.35	9.67	17.39	4.21	58.25	4.74
Individual processes:						
Conventional dry grind, no oil skimming	27.30	10.43	n/a	n/a	n/a	n/a
Conventional dry grind, with oil skimming	27.40	8.90	n/a	n/a	n/a	n/a
Wet milling	n/a	n/a	17.39	4.21	58.25	4.74
Scenario 5: Full starch conversion	29.10	10.28	17.39	4.21	58.25	4.74
Scenario 6: Complete fiber conversion plus full starch conversion	28.58	10.10	17.39	4.21	58.25	4.74

^a^Expressed on a commercial or ‘as is’ basis. Dry matter contents of 89.31%, 87.13%, and 90.04% for DDGS, CGF, and CGM, respectively [40]. ^b^Weighted industry averages.

**Table 5:** Ethanol and co-product outputs associated with ethanol processing scenarios, assuming 86% dry grind with 50% skimming oil and 14% wet milling

	**Ethanol yield, L/t (gal/bu)**	**DDGS yield, kg/t (lb/bu)**^**a**^	**CGF yield, kg/t (lb/bu)**^a^	**CGM yield, kg/t (lb/bu)**^**a**^	**Dry grind oil recovery, kg/t (lb/bu)**
Baseline^b^	410.3 (2.759)	310.1 (17.44)	229.3 (12.88)	49.77 (2.80)	2.145 (0.12)
Individual processes:					
Conventional dry grind, no oil skimming	414.1 (2.785)	312.2 (17.56)	n/a	n/a	0
Conventional dry grind, with oil skimming	414.1 (2.785)	308.0 (17.32)	n/a	n/a	4.288 (0.24)
Wet milling	386.6 (2.600)	n/a	229.3 (12.88)	49.77 (2.80)	n/a
Scenario 5: Full starch conversion	420.7 (2.829)^#^	291.2 (16.38)	229.3 (12.88)	49.77 (2.80)	2.145 (0.12)
Scenario 6: Complete fiber conversion plus full starch conversion	457.6 (3.078)^#^	226.3 (12.67)	229.3 (12.88)	49.77 (2.80)	2.145 (0.12)

^a^Commercial or ‘as is’ basis. Dry matter contents of 89.31%, 87.13%, and 90.04% for DDGS, CGF and CGM, respectively [40]. ^b^Weighted industry averages. DDGS, distillers dried grains with solubles; CGF, corn gluten feed; CGM, corn gluten meal.

This effect is even more exaggerated in Scenario 6, which features fermentation technology to convert C5 and C6 sugar to ethanol, converting not only residual starch but pericarp and endosperm fiber fractions as well. With complete fiber (and starch) conversion, the ethanol yield per bushel of processed corn increases from 2.759 to 3.078 gallons, compared with baseline ethanol processing efficiencies. Correspondingly, the quantity of DDGS produced as co-product decreases from 17.44 to 12.67 pounds per bushel of processed corn (Table 5). Similar to Scenario 5, Scenario 6 presents an interesting trade-off: an overall 11.6% increase in ethanol yield per bushel of corn for a 27.4% decrease in the quantity of DDGS produced per bushel of corn. The decrease in feed co-products results in less corn and soybean meal being substituted, which in turn raises the land area attributed to corn ethanol. In general, land area for corn ethanol is higher when the system extracts more ethanol per bushel of corn.

With both full starch and complete fiber conversion, there is no advantage in terms of reduced land usage attributed to corn ethanol production, even after 15 years. In 2026, 13% (Scenario 5) and 14% (Scenario 6) of US corn-producing land is attributable to corn ethanol, compared to 13% (Scenario 1) without enhanced technological efficiency in ethanol processing to output more ethanol from every bushel of corn processed. The reverse side is that the land usage attributed to corn ethanol is negatively impacted only slightly from as much as an 11.6% increase in ethanol output as corn yields increase to 2026 levels.

Scenario 7 demonstrates the impact of a shift in feeding value for DDGS, CGF, and CGM, with substitution for corn falling from 71% to 65% in livestock diets and substitution for soybean meal increasing from 29% to 35%. This shift reflects a change in the allocation of the feed co-products across livestock types. Specifically, non-ruminants and dairy cattle consume higher proportions of ethanol co-products, whereas beef cattle consume less (Table 6). The shift reflects a tension between offsetting corn grain or soybean meal consumption when feeding ethanol co-products to livestock. Feeding more DDGS to monogastric animals not only has the benefit of replacing more high cost protein from soybean meal with a lower cost alternative, but land usage attributed to corn ethanol production is dramatically reduced as well. This effect is most pronounced with 2026 estimates: only 1.2 million of the total US corn acreage (1%) is attributed to corn ethanol production without oil replacement (Table 1). However, with the shift to 35% replacement of soybean meal in livestock diets, land area attributed to corn ethanol rises to 10.3 million acres (12%) due to higher oil replacement demands.

**Table 6:** Percentage of feed usage domestically of distillers dried grains with solubles produced from corn grain directed to dry grind processing (4.306 billion bushels) by livestock type with 71%:29% (baseline) and 65%:35% (Scenario 7) corn-to-soybean substitution ratios

	**Percentage of total distillers dried grains with solubles usage (%)**	
**Livestock type**	**71%:29% substitution ratio**	**65%:35% substitution ratio**
Beef cattle	50.4	30.0
Dairy cattle	33.5	47.3
Pigs	9.1	12.8
Poultry	7.0	9.9

Interestingly, feeding a higher percentage of co-products to monogastrics and dairy cattle provides a negative feedback force that reduces the land usage attributed to corn ethanol production. Land usage falls in the terminal period by 0.7 million acres or one percentage point when comparing Scenario 7 with Scenario 1, assuming oil replacement. This occurs because offsetting a low yield crop like soybean through co-product feeding reduces land usage more than a high yield crop like corn. However, this difference would diminish with increases in soybean yield (which were fixed in this analysis), perhaps making corn and soybeans more comparable as land use alternatives in the corn ethanol system.

## Conclusions

Corn grain yield has a profound impact on estimates of land area attributable to corn ethanol production. In 2011, 25% of the acreage used for US production of corn grain was devoted to ethanol fuel production based on the historic corn yield for the year and accounting for replacement of soybean oil with the reduced demand for soybean production. Assuming reasonable increases in corn grain yield with anticipated new yield technologies coming into play in the next 15 years, this percentage could be reduced by nearly half to 13%. Even assuming the most conservative estimate of corn yield growth, that is, Scenario 4, land area attributed to ethanol production drops to 19%. The high rate of technological change in corn production combined with the strong linkage between yield and land use requires biofuel life-cycle analysts to include insightful estimates of yield as well as yield dynamics within models.

Co-product utilization is a powerful force reducing the land usage attributable to corn ethanol in the US corn ethanol system. Thus, the system complementarity between fuel production and livestock nutrition improves because ethanol co-products provide a negative feedback substituting for corn and soybean meal, which in turn reduces land demand for corn and soybean production. System complementarity may be an important element for biofuel life-cycle analysts as they think about the full impacts of the systems under study.

Substitution of soybean meal through co-product feeding removes oil from the market. Accounting for that oil through replacement significantly increases estimates of land area estimates attributed to corn ethanol production. This positive feedback force is especially acute because oil production (soybean or canola) is relatively land intensive compared to starch production (corn). This effect could be ameliorated by substituting an oil crop with a high land use efficiency. We use canola in our model, one of the highest oil-yielding crops on a per acre basis. But perhaps on the horizon, through plant breeding, more superior canola cultivars or other crop alternatives might emerge.

By contrast, improved efficiency in ethanol processing through anticipated fermentation technologies that increase ethanol output has little impact on land area attributed to corn ethanol over time. This is due to the trade-off between volumes of ethanol and co-product outputs, where small improvements in ethanol-processing efficiency significantly reduce amounts of co-product. Thus the need for less corn, and corn land, through advanced processing technologies is offset by reduced negative feedback because there is less co-product to substitute for corn and soybean meal in livestock feeding. The ethanol/DDGS trade-off in dry grind processing may warrant greater scrutiny by ethanol processors from an economic standpoint to maximize returns.

The anticipated change in the overall corn and soybean meal substitution ratio in DDGS livestock feeding only slightly decreases the amount of land area attributed to corn ethanol production. A sizeable (11%) difference in land area with oil replacement versus without oil replacement signifies the importance and prominence of oilseeds to US agriculture.

This analysis demonstrates clearly that, while 40.5% of harvested corn grain in the US goes to ethanol production, the percentage of total corn acreage attributed to corn ethanol production is much less. The estimate of land area attributed to corn ethanol production continues to fall as corn yields increase over time and the 15 billion gallon cap on corn ethanol is reached. As greater output is achieved in corn grain production, the land base required to produce a given volume of grain is reduced. This could translate to either less acreage to produce 15 billion gallons of corn ethanol or an enlarged market opportunity from the same allocation of land. Further research would be helpful to better specify both the demand side impacts of advancing technology, as well as the feedback processes affecting technology research and development investments.

The results challenge other findings about land use and indirect effects on land use change associated with corn ethanol at current and future production levels (for example, see [14-20]) and may be useful in establishing parameters for land use in models that consider a broader view of the biofuel arena. Furthermore, the results cited herein support inferences of others about US agricultural land productivity (for example, [11,12,21]) and the capacity to meet increasing demand in the future. Finally, this analysis also highlights the importance of accounting for technological change to better understand how a particular biofeedstock impacts various aspects of the whole biofuel picture. Technological change challenges discipline specialists when they attempt to analyze an explicitly multidisciplinary system involving numerous technologies outside of their domain expertise. We would argue conceptually, and from our experience coming together on this project, that multidisciplinary teams are essential when researchers choose to explore the macro biofuels system.

## Methods

Technological changes anticipated in each of the three disciplinary areas are described in detail below and related to the seven defined scenarios. In addition, details of the model of the US corn ethanol system created to simulate effects on land usage attributed to corn ethanol production are outlined. Inputs and outputs of this model are varied depending on the scenario.

### Model

The systems dynamic package, STELLA [22], was used to model current practices and the effects of technological changes related to corn yield, ethanol processing, and livestock feeding on US land usage attributed to corn ethanol production. Our approach was not to mimic real life because the agricultural system in which we are working is extremely complex. Instead, focusing on the three factors of interest, we evaluated critical drivers identified through dialogue with scholars and industry to develop a model that is simultaneously manageable and useful for analysis.

**Figure 2:**
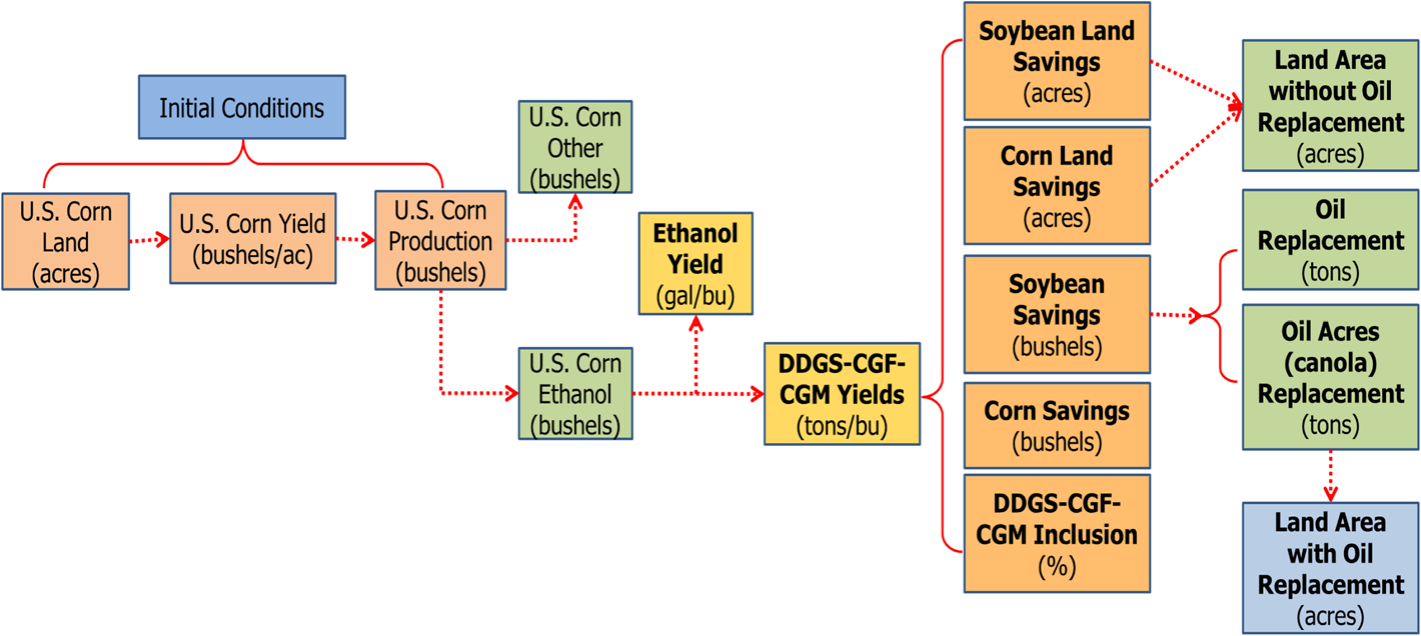
Overview of the model to simulate land area attributed to corn ethanol production.

Our simple model (Figure 2) is centered on the US agricultural land base, the proportion of the US corn crop processed for ethanol, soybean and canola crops, livestock population and feeding practices, a 15 billion gallon corn ethanol industry, and a time frame from 2011 to 2026. Estimates of land usage attributed to corn ethanol production are a function of time (future prediction) as well as the variables defined by the scenario. Variables in the model include average US yields of harvested corn grain; ethanol production volumes; availability of corn ethanol processing co-products including DDGS, CGF, and CGM; and inclusion rates of corn grain, soybean meal, and corn ethanol co-products in diets fed to poultry, pigs, beef cattle, and dairy cattle. The model is designed to track both positive and negative feedback^a^ processes, for example, the availability of skimmed oil from dry grind processing and replacement of the soybean oil from acres of soybean for meal that are displaced by DDGS feeding. Thus, the model is sensitive to variable inputs as well as internal system feedbacks given the parameters and assumptions specified.

The model produces estimates of land usage attributed to corn ethanol under two conditions: without accounting for replacement of soybean oil from the displaced acres of soybean for meal; and accounting for replacement of soybean oil. With the latter, canola is given as the example of an oil crop replacement for soybean, although others or a mixture of oil crops could be envisioned. Trending on the side of conservatism and with the intent to highlight the three factors under study, yields of soybean and canola crops were held at 2011 levels in the model. Also, the model does not reflect the extra canola meal feed produced through canola replacement of soybean acreage. Fixed in the model are US average soybean yields of 41.9 bu/A (2011 level) [23]; an average 78% yield of soybean meal from soybeans processed [24]; and US canola grain yields at 1,713 pounds per acre (2010/2011 level) [25], resulting in a canola oil yield of 753.7 pounds per acre given an average of 44% oil composition.

The dynamic model computes estimates of land use attributed to corn ethanol in 2011 and 2012 according to actual corn grain acreage and yield figures. The model estimates land use attributed to corn ethanol over the next 15 years (to 2026) under various assumptions related to the above variables and their interactions as defined by the specific scenario. In addition, the model identifies the year in which the 15 billion gallon cap is reached. The model commences with the corn production acreage in the US as a fixed land base according to 2011 levels (83.98 million acres), with harvested grain flowing to either processing for ethanol or other uses (for example, exports, feed). Ethanol production is capped over the 15-year period at no more than 40.5% of the US corn crop or 15 billion gallons of ethanol (or 14.71 billion gallons before denaturation at an assumed 2.2% denaturation rate). This prohibits a greater portion of the total corn grain harvested in any given year that is directed to corn ethanol exceeding either 2011 levels or US policy standards. Holding these factors at a steady state facilitates focus on the factors under investigation.

Over the 15-year window, the seven scenarios build on a baseline involving estimates of US corn grain yields forecasted as a result of a moderate effect of technological influence along with 2011 current practices in ethanol processing and livestock feeding. Only the specified effect (for corn grain yield, ethanol processing, or livestock feeding) is changed for each scenario.

Corn yield forecasts are based on historical trends and in-depth industry analysis and input. Ethanol processing forecasts are based on outputs reported by Mueller [26] and other industry data [27]. Livestock feeding forecasts were linearly extrapolated from the historical trend. Details of the baseline and specified scenarios for corn production, ethanol processing, and livestock feeding are provided in the following sub-sections.

### Crop yield forecasts

Technologies in the area of corn production focus on increases in grain produced per unit of land through both genetic and agronomic improvements. Genetic improvements can be partitioned to identify increases due to quantitative incremental gains through breeding, increased efficiency realized in the seed product pipeline (that is, speed to market) due to new technological innovations, and biotechnology traits (traits manifest through genetic modification) [28]. Agronomic improvements focus on ways to maximally leverage the crop genetic potential through cultural production practices, including spatial arrangement of plants in the field, pre-emptive measures for plant health, soil management and fertility, and seed treatments. In addition, global positioning systems technologies enable increasingly precise application of added nutrients and variable spacing of seed to maximize response to the micro-environment defined by soil type, topography, and other factors.

To derive Scenario 1 yield estimates (Table 2), actual corn yields for 2011 and 2012 were used [1]. Corn grain yields forecast for 2013 through to 2026 in keeping with historical trends, anticipated commercial launch dates, and farmer adoption of technologies, with the following assumptions:

• Based on trends observed from 1930 (development of hybrid corn and adoption replacing open-pollinated varieties) through 2012 [1], an average gain of 1.8 bu/A has been realized per year. This gain coincides with realized gains per year of 1.81 bu/A based on USDA average yields during the single cross hybrid era from 1960 to 1995, before genetically modified traits were commercialized in corn [29]. Thus, an average yearly gain of 1.8 bu/A is assumed to carry forward through 2026 (Table 2) from its historic trend line.

• Advanced breeding technology facilitating genomic-based approaches to choosing parents and identifying superior progeny in breeding populations as well as means to accelerate the breeding process to accumulate genetic gains more rapidly (for example, doubled haploid technology and associated breeding strategies) are expected to contribute additional gains of 1 bu/A per year. Based on widespread adoption of advanced technologies in the early to mid-2000s by the larger seed companies, this step change is likely to be fully realized beginning in 2015, with the launch of corn hybrids developed with these innovations (Table 2). A phase-in period is assumed, with the portion of new hybrid offerings developed using advanced technologies estimated at 25% in 2013 and 50% in 2014.

• Three releases of biotechnology traits are anticipated by 2026, each involving combinations (that is, trait stacks) of novel or improved biotechnology traits, with each combination delivering an estimated yield increase of 10 bu/A (Table 2). The leading biotechnology trait provider in corn, Monsanto Company, together with trait discovery partner, BASF, anticipates that by 2020 new corn hybrids will include >10 biotechnology traits [30] and as many as 20 by 2030 [31]. Monsanto refers to ‘yield and stress packages’, with enhancements to first-wave trait releases to follow in subsequent trait packages. The package of biotechnology traits would produce a step change in yield by preserving genetic potential through pest and stress tolerances or resistance, enhancing genetic potential, or improving efficiency in the plant utilization of essential requirements such as water and nitrogen. Biotechnology traits in phase III and phase IV stages of development by 2012 were considered to be 2 to 5 years away from market launch [32], for example, drought tolerance I, high yield corn, and CRW (corn rootworm) III featuring RNAi mode of action [33,34]. Biotechnology traits in phase II stage of development by 2012 were considered to be 3 to 7 years away from market launch [32], for example, drought tolerance II, nitrogen use efficiency, and ECB (European corn borer) III [33,34]; this would constitute a second-wave package. The third-wave package of traits is presumed to include next-wave enhancements of the traits in the earlier packages. No phase-in period is accounted for, that is, the effect is not included until the years forecasted for maximal adoption of each release: 2018, 2022, and 2025. Other biotechnology trait providers may also contribute to trait technology to the marketplace; however, product pipeline information from other potential providers was not available publicly for this analysis. Because biotechnology traits are licensed across seed companies in the industry, market penetration does not depend on the market share of any one seed company.

• Agronomic improvements are anticipated in three waves by 2026, each accounting for a 3 bu/A yield increase (Table 2). Seed companies are working with manufacturers of farm machinery to create ‘smart systems’ to maximize yield through best possible agronomic conditions. For example, Monsanto Company plans the market introduction of IFS (Integrated Farming Systems) I, featuring variable rate planting, as early as 2014 [33,34]. A second wave of agronomic improvements is anticipated, with prescription placement for fertility and water added to IFS II [33]. The development of agricultural biologicals that boost the efficiency of pest controls such as BioDirect™ Technology by Monsanto [33] may factor into a third wave of agronomic improvements. Conservative estimation of yield impact forecasts effect step changes at maximal technology adoption in 2016, 2020, and 2024, with no phase-in period.

Scenario 1 corn yield forecasts serve as a baseline to represent grain input to scenarios that do not consider grain yield (that is, Scenarios 5 to 7).

Scenarios 2 and 3 were developed following Lywood *et al*. [9], based on 10% decrease and 10% increase of Scenario 1 estimates, respectively (Table 3). Note that projections begin in 2013 because actual data are used to represent 2011 and 2012 yields. In contrast to the yield technology scenarios, Scenario 4, which reflects little/no yield gain from technological advancements, was developed using USDA long-term Projection figures that forecast corn yield increases at 2.0 bu/A through 2021 [35]. Extrapolating through 2026, corn yields are predicted to reach a level of 192.0 bu/A with Scenario 4 (Table 3).

Future genetic improvement could be directed to a greater quantity of starch in corn and possibly a higher quality of starch for ethanol production. However, use of such corn grain for improved ethanol yield would require identity preservation of grain destined for ethanol production; such a scenario for corn ethanol raw material supply is unlikely to be widely implemented in the next 15 years [36]. Therefore, this condition was not modeled.

### Forecasted changes in ethanol production

There are two methods used to produce ethanol from corn grain: dry grind and wet milling, which account for 86% and 14% of total US production, respectively [26]. Technologies in corn ethanol production focus on increasing efficiencies, leading to greater ethanol output. Advancements, mainly pertaining to dry grind, reflect process modifications that have been developed by researchers but are yet to be adopted at a large scale [37-43]. Enzyme products are being tested and adopted that make more use of the starch in the corn kernel, serving to increase ethanol yields and reduce the residual starch content of the DDGS. Enzymes have been used at a commercial scale that aid liquefaction and saccharification, thus improving the yeast’s ability to convert glucose to ethanol [44-47]. Furthermore, experimental enzymes show promise in converting the cellulose and hemicellulose in the kernel to increase ethanol yields as well as reduce these fiber compounds in the DDGS co-product [48-50].

**Figure 3:**
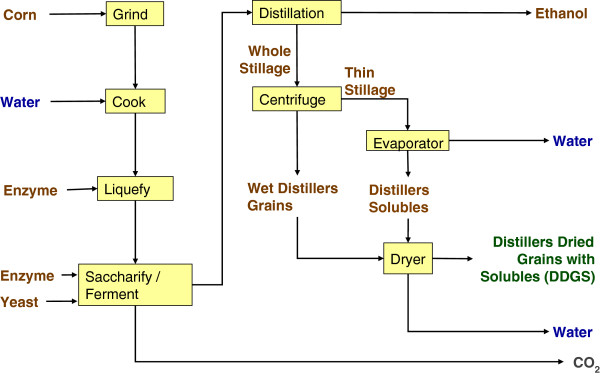
Conventional dry grind process for production of ethanol and distillers dried grains with solubles.

**Figure 4:**
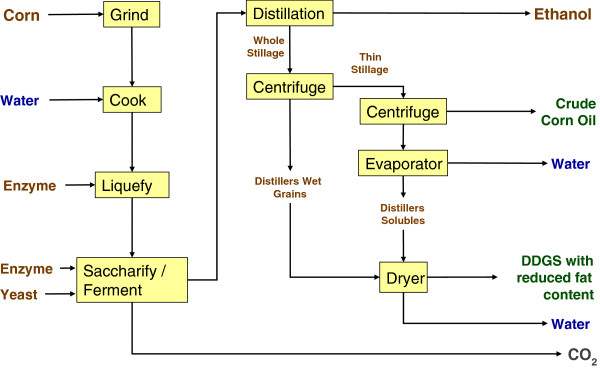
Dry grind process with oil recovery for production of ethanol, oil, and reduced fat distillers dried grains with solubles.

**Figure 5:**
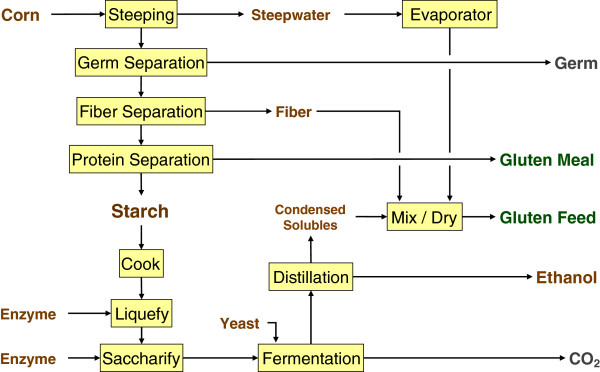
Wet milling process for production of ethanol, germ from which oil and germ meal are recovered, corn grain feed, and corn grain meal.

Dry grind and wet milling differ with respect to types and amounts of process outputs. The primary outputs from the dry grind process are ethanol, DDGS, and oil when skimming is practiced (Figures 3 and 4). With wet milling, higher-value co-products result, in the form of CGF, CGM, and corn oil (from the germ) suitable for human use; DDGS is not produced (Figure 5). DDGS, CGF, and CGM are relatively high in protein and thus directly supplant soybean meal, as well as corn grain, in livestock diets. Among dry grind ethanol plants, an estimated 50% practice oil skimming to recover crude oil that is mainly utilized for biodiesel production [26]. In this way, the skimmed oil competes with soy oil as a raw bioenergy feedstock. As soybean meal is supplanted in livestock diets by DDGS from corn ethanol, the soy oil produced along with soybean meal must be accounted for as well. Furthermore, oil skimming has an effect on DDGS composition, lowering the fat (energy) component as well as the protein content. The composition of feed co-products, that is, protein and fat content, depends on the ethanol processing method (Table 4).

A weighted average of ethanol and co-product outputs was computed and used to formulate a baseline to represent ethanol processing in scenarios other than Scenarios 5 and 6 that feature effects of technological changes to the ethanol process (Table 5). Baseline assumptions include:

•A weighted industry yield of 2.759 gallons per bushel reflects dry grind and wet milling ethanol yields of 2.785 and 2.600 gallons per bushel, respectively, with dry grind plants making up 86% of ethanol capacity and wet milling plants 14%.

• Oil skimming, which is done at 50% of dry grind plants, leads to 0.24 pounds of oil per bushel, decreasing DDGS yield to 17.32 pounds per bushel at 89.31% dry matter content (that is, 15.45 pounds dry basis per bushel). Plants that do not skim have DDGS yields of 17.56 pounds (as is) per bushel [26]. Thus, the weighted dry grind average DDGS yield is 17.44 pounds (as is) per bushel.

• Wet milling co-products CGF and CGM represent 23% and 5% output from each bushel of corn processed for ethanol, respectively.

• Based on the current US ethanol production capacity of 14.71 billion undenatured gallons per year [27] and assuming a 50% adoption rate for skimming oil at a rate of 0.24 pounds of oil per bushel, oil production from skimming is estimated at 545.3 million pounds. This can be converted for use as biodiesel at a rate of 9 pounds of biodiesel per 10 pounds of oil, for a total biodiesel volume from skimmed oil of 66.68 million gallons (which is well below the US mandated biodiesel production of 1 billion gallons). It was assumed that biodiesel gallons from skimmed oil would replace gallons produced using soy oil; thus, skimmed oil feeds back into the model to reduce the amount of oil replacement acres by canola. Because oil skimmed from the dry grind process is not economical for use in human food, this prevents the use of some of the soy oil for non-food use.

Scenario 5 anticipates a technological improvement in the dry grind process that allows for fermentation of all starch in the corn grain to ethanol, providing 2.5% more ethanol output per bushel of corn while reducing DDGS production by 6.1%. This scenario is referred to as ‘full starch’. At present, a percentage of starch remains unconverted in the DDGS following fermentation, as indicated by average starch content (7.54% starch (dry basis)) of DDGS samples from the National Research Council [51]. If a greater percentage of starch could be converted into ethanol, ethanol yields would increase, DDGS output per bushel of corn would decrease, and DDGS composition would be altered. Dry grind processors continue to use new equipment, enzymes, and process designs in an effort to reduce residual starch content of DDGS. We assume this improved technology would be implemented at all dry grind facilities. Conservatively estimating that 6% residual starch (dry basis) or other convertible sugars in DDGS from dry grind are converted (sources such as the National Research Council report DDGS starch contents of 7.5% to 10.8% (dry basis) [51]), the additional ethanol yield with full starch implementation is estimated at 0.081 gallons per bushel. In Scenario 5, we accounted for changes in yield and composition of DDGS but assumed no impact to CGF or CGM because of the comparatively small fraction of materials in the livestock feeding system and the minimal impact anticipated. Thus, the full starch scenario involves only changes to dry grind co-product outputs; compositions of CGF and CGM were assumed to be unchanged. With more complete starch conversion during dry grind, dry grind ethanol yield increases to 2.866 gallons per bushel, and the improved aggregate industry yield is 2.829 gallons per bushel (Table 5; see Additional file 1 for calculations).

Concomitant with ethanol yield increase, the amount of DDGS produced per bushel decreases due to the complete fermentation of starch. The adjusted rate of DDGS production due to full starch conversion, assuming 50% adoption of oil skimming by the dry grind industry, is 16.38 pounds of DDGS per bushel of corn (Table 5; see Additional file 1 for calculations). At the same time, protein content per bushel of corn increases to 29.10%, although total protein in the DDGS produced per acre of corn remains the same; likewise, fat (oil content) increases per bushel of corn to 10.28% (Table 4).

Scenario 6 anticipates another technological improvement in fermentation in the dry grind process to facilitate conversion of fiber fractions (C5 and C6 sugars) to ethanol, thus leading to 11.6% more ethanol output per bushel of corn while concomitantly reducing DDGS output 27.4%. This scenario, referred to as ‘complete fiber’, anticipates conversion of fiber portions of the corn grain that are currently unfermented, in addition to conversion of residual starch (as in Scenario 5). Dien *et al.* document conversion of C5 and C6 sugars to ethanol using conventional yeast and a bacterial strain (*Escherichia coli* FBR5) [48]. More recently, Ha *et al*. [52] and Bera *et al*. [53] document the conversion of C5 sugars to ethanol with new fermentation organisms. The fermentation included residual starch along with pericarp and endosperm fiber fractions. Dien *et al*. reported that use of two types of glucose-consuming organisms increased ethanol yields by 13.3% [48].

In dry grind processing, the 13.3% increase in ethanol yields is accompanied by changes in DDGS yields and composition. Because ethanol is a high-value product, it is assumed that 100% of dry grind producers would adopt this technology (although we do not expect the number and proportion of dry grind facilities to be affected by this technology). The wet milling facilities were not anticipated to adopt this technology because feasible production of CGF relies on a source of fiber that is mixed and dried with process streams such as steepwater and fermentation solids (distillers solubles). In wet milling, fiber is needed as a method to allow removal of steepwater and fermentation solids from the process. Without the fiber stream, drying these solids would not be economical.

The 13.3% increase in ethanol yield with the complete fiber scenario translates to an additional ethanol yield of 0.370 gallons per bushel for dry grind, bringing the aggregate industry yield to 3.078 gallons per bushel (Table 5; see Additional file 1 for calculations). Concomitant with ethanol yield increase, the composition of DDGS output in dry grind processing is altered with complete fiber conversion compared to the baseline. Protein content per bushel of corn increases to 28.58%; likewise, fat (oil content) increases per bushel of corn to 10.10% (Table 4). It could be anticipated that protein and oil contents for Scenario 6 would be much higher than Scenario 5. However, Scenario 5 assumes a 6.1% decrease in the DDGS yield, whereas Scenario 6 is based on actual conversion rates from Dien *et al.*[48]. With the decreased amount of DDGS produced per bushel, the adjusted rate of DDGS production due to complete fiber conversion, assuming continued 50% adoption of oil skimming by the dry grind industry, is 12.67 pounds of DDGS per bushel of corn (Table 5; see Additional file 1 for calculations).

### Changes in the usage of co-products for livestock feed

Technologies in the area of livestock feeding and nutrition for poultry, pigs, beef cattle, and dairy cattle anticipate increased usage of ethanol co-products, mainly DDGS, by specific groups of livestock. Altered usage of DDGS assumes compliance with dietary nutrition consistent with maintaining high meat and milk quality.

In 2011, it is estimated that a total of 127.440 million metric tons (5.007 billion bushels) of corn was directed to ethanol production annually [25], with 86% (109.598 million metric tons; 4.306 billion bushels) being used in dry grind processing and 14% (17.842 million metric tons; 700.917 million bushels) being used in wet milling. Given a weighted average of 310.1 kg of DDGS produced per metric ton of corn grain in the dry grind process (Table 6), a total production of 34.091 million metric tons of DDGS resulted (Table 7). In addition, 4.098 million metric tons of CGF and 0.891 million metric tons of CGM were generated as a result of production of ethanol from corn in the wet milling process, assuming that 23% of the corn grain will end up in CGF and 5% of the grain will end up in CGM. Thus, an estimated total of 39.080 million metric tons of DDGS, CGF, and CGM are produced annually from corn ethanol processing. It is assumed that of the DDGS produced, 9 million metric tons are exported, and the remaining 25.091 million metric tons are used domestically.

**Table 7:** Substitution of corn and soybean meal by distillers dried grains with solubles, corn grain feed, and corn grain meal produced from 5.007 billion bushels (127.440 million metric tons) of corn grain associated with 71%:29% substitution ratio overall

		**Substitution (%)**		**Substitution tonnage (millions)**	
**Co-product**	**Million tons**	**Corn**	**Soybean meal**	**Corn**	**Soybean meal**
Export	9.000	51.2	48.8	4,610	4,390
Domestic use	25.091	75.8	24.2	19.020	6.071
Beef usage	12.646	100	0	12.646	0
Dairy usage	8.405	47.0	53.0	3.951	4.455
Pig usage	2.283	60.0	40.0	1.370	0.913
Poultry usage	1.756	60.0	40.0	1.054	0.703
Domestic dairy, pigs, and poultry	12.445	51.2	48.8	6.374	6.071
Distillers dried grain with solubles domestic plus export	34.091	69.3	30.7	23.630	10.461
Corn grain feed	4.098	100	-	4.098	-
Corn grain meal	0.891	-	100	-	0.891
Total substitution	39.080	71.0	29.0	27.728	11.352

Because DDGS, CGF, and CGM supplants different amounts of corn and soybean meal in diets fed to different groups of animals, it is necessary to know the approximate market share for each group of animals for which DDGS is used. There are three recent estimates for the proportion of domestic DDGS fed to beef cattle, dairy, pigs, and poultry [54-56]. The average of these three estimates is 50.4% to beef cattle, 33.5% to dairy cattle, 9.1% to pigs, and 7.0% to poultry (Table 6). Based on these percentages, the total usage of DDGS for each group of animals can be calculated (Table 7).

Inclusion of DDGS, CGF, and CGM in livestock feeding regimes supplants corn or soybean meal in diets for beef cattle, dairy, poultry, and livestock. At present, DDGS replaces approximately 60% corn and 40% soybean meal in the feeding of pigs and poultry [54,57], supporting a 1:1 substitution rate [58,59]. DDGS replaces various amounts of corn and soybean meal in diets for ruminant animals. The rate of substitution per species depends on the requirement for protein and energy. It is assumed that DDGS fed to beef cattle replaces no soybean meal and only corn because beef cattle are usually fed only limited quantities of soybean meal due to their relatively low requirement for protein and because protein equivalents can be obtained less expensively from other ingredients. Thus, it is assumed that DDGS included in diets fed to beef cattle replaces corn at a 1:1 rate, although it is acknowledged that substitution rates of 1.1:1 or 1.2:1 have been proposed [54]. However, currently there are very limited biological data to support substitution rates greater than 1:1 and in the present calculations, the 1:1 rate is used to make sure the DDGS is not overvalued.

It is also assumed that the CGF produced from the wet milling industry is fed exclusively to beef cattle and that it replaces corn on a 1:1 basis and that no soybean meal is replaced by CGF. By contrast, DDGS fed to dairy cows replaces 47% corn and 53% soybean meal and DDGS fed to pigs and poultry replaces 60% corn and 40% soybean meal. As a consequence of these substitution rates, more soybean meal is replaced if DDGS usage is shifted from beef cattle to dairy, pigs, or poultry. It is also assumed that the 9 million metric tons of DDGS that are exported are fed to dairy, pigs, and poultry and that the substitution rates for corn and soybean meal for the exported DDGS are similar to the replacement rates for the domestically used DDGS that is fed to dairy, pigs, and poultry (Table 7). Finally, it is assumed that the CGM that is produced from the wet milling industry is used exclusively in dairy feeding where it replaces soybean meal at a 1:1 rate. Under these assumptions, the total substitution of corn and soybean meal can be calculated.

Overall, 27.728 million metric tons of corn and 11.352 million metric tons of soybean meal are replaced across livestock diets, reflecting a substitution ratio of 71% corn to 29% soybean meal in feeding of DDGS, CGM, and CGF (Table 7). This ratio serves as a baseline to represent livestock feeding utilization of corn ethanol co-products in scenarios other than Scenario 7, which reflects an adjusted ratio based on technological changes.

Scenario 7 anticipates a modified overall substitution ratio of corn to soybean meal due to a shift in the utilization of DDGS in feeding diets among livestock types. Because economics favor replacement of soybean meal rather than corn (amount of protein on a per ton basis), it is expected that an increased proportion of DDGS will be consumed by dairy, pigs, and poultry in the future.

Dairy cattle are expected to be a primary target for increased rates of DDGS inclusion in the diet; dairy cattle require a high amount of protein, which is largely provided by soybean meal at present, and can also handle the fiber load. DDGS can be used in diets for dairy cows by at least up to 20% and often up to 30% on a dry matter basis without changing animal performance [60,61]. Furthermore, use of DDGS for dairy favors the lower fat content of DDGS from dry grind ethanol plants that skim oil (Table 4); thus, oil skimming may promote a higher rate of inclusion of DDGS in diets fed to dairy cows [61]. Provided that diets are correctly formulated, there are no indications that milk composition will be changed or that the value components in milk will be reduced [62,63].

With the increased awareness of the benefits of DDGS in diets fed to pigs and poultry and the economic competitiveness of DDGS relative to soybean meal, it is also likely that the penetration of DDGS in the swine and poultry feed markets will increase. This can be easily accomplished without exceeding the maximum recommended rates for inclusion of DDGS in diets fed to pigs or poultry. DDGS can be included in diets for pigs at levels of at least 20% without changing the composition or the nutritional value of the meat that is produced [59,64-67]. Likewise, for poultry, DDGS can be included in the diets by at least 10% to 15% without reducing product quality [68-70]. Because DDGS penetration can be increased greatly without exceeding these thresholds, it is possible to increase DDGS utilization in diets fed to pigs and poultry without negatively impacting pork or poultry meat quality. Furthermore, balancing of DDGS with specific indispensable amino acids will make DDGS more usable in the feeding of pigs and poultry, and use of specific microbial enzymes such as xylanase and phosphatases in livestock diets containing DDGS may help increase the energy and phosphorus value of DDGS. By feeding more DDGS to pigs and poultry, these livestock types will consume an overall greater share of the total DDGS produced.

With greater use of DDGS in feeding of dairy cattle, pigs, and poultry, a decline in the proportional usage by beef cattle is anticipated. In Scenario 7, it is therefore estimated that consumption of DDGS by beef cattle is reduced from the current level of 50.4% to only 30% of total DDGS production, whereas dairy cattle, pigs, and poultry will increase consumption to 47.3%, 12.8%, and 9.9% of the produced DDGS, respectively (Table 6). Reflecting the shift in usage among livestock types, Scenario 7 depicts DDGS substitution of 65% corn and 35% soybean meal across livestock diets (Table 8).

**Table 8:** Substitution of corn and soybean meal by distillers dried grains with solubles, corn grain feed, and corn grain meal produced from 5.007 billion bushels (127.440 million metric tons) of corn grain associated with a 65%:35% substitution ratio overall

		**Substitution (%)**		**Substitution tonnage (M)**	
**Co-product**	**Million tons**	**Corn**	**Soybean meal**	**Corn**	**Soybean meal**
Export	9.0	51.2	48.8	4,610	4,390
Domestic use	25.091	75.8	24.2	19.020	6.071
Beef usage	7.527	100	0	7.527	0
Dairy usage	11.868	47.0	53.0	5.578	6.290
Pig usage	3.212	60.0	40.0	1.927	1.285
Poultry usage	2.484	60.0	40.0	1.490	0.994
Domestic dairy, pigs, and poultry	17.564	51.2	48.8	8.995	8.568
Distillers dried grain with solubles domestic plus export	34.091	62.0	38.0	21.132	12.959
Corn grain feed	4.098	100	-	4.098	-
Corn grain meal	0.891	-	100	-	0.891
Total substitution	39.080	64.6	35.4	25.231	13.850

### Endnote

^a^We define feedback drawing on terminology from the field of System Dynamics, http://www.systemdynamics.org/DL-IntroSysDyn/feed.htm. Positive feedback causes systems to grow or expand, and negative feedback causes decline or contraction. For example, in our case, a positive feedback effect results when a perturbation to the system increases land area, such as with higher ethanol yields, whereas a negative feedback effect, such as greater livestock feeding with DDGS, reduces land area.

## Abbreviations

bu/A: bushels per acre; CGF: corn gluten feed; CGM: corn gluten meal; db: dry basis; DDGS: distillers dried grains with solubles; USDA: United States Department of Agriculture.

## Competing interests

The authors declare that they have no competing interests.

## Authors’ contributions

RM conceived the study. All authors were involved with designing the study. RM, KR, and HS performed the microanalyses. PG created the model and conducted the system analysis. RM and PG drafted the manuscript, with KR and HS contributing key portions. All authors read and approved the final manuscript.

## Supplementary Material

Additional file 1 **Additional documentation is available that shows the calculations related to ethanol processing used to generate estimates for Tables** 4**and**5**.** See PDF file: Additional information: Calculations related to ethanol processing. Click here for file
