# Supplementary material for: Land usage attributed to corn ethanol production in the United States: sensitivity to technological advances in corn grain yield, ethanol conversion, and co-product utilization
Source: Biotechnol Biofuels. 2014 Apr 12;7:61. doi: 10.1186/1754-6834-7-61 (PMC4022103; doi:10.1186/1754-6834-7-61)
Supplement: Additional file 1 — Additional documentation is available that shows the calculations related to ethanol processing used to generate estimates for Tables 4and5. See PDF file: Additional information: Calculations related to ethanol processing. [file 1754-6834-7-61-S1.docx]

**Land usage attributed to corn ethanol production in the United States: sensitivity to technological advances in corn grain yield, ethanol conversion, and co-product utilization**

R. H. Mumm, P. D. Goldsmith, K. D. Rausch, and H. H. Stein

**Additional information: Calculations related to ethanol processing***

*Baseline*

If 2.6 gal per bushel is assumed for the wet milling process and 2.785 gal per bushel for the dry grind process as per [Mueller](#_ENREF_6) [26], then the aggregate industry yield can be calculated based on 14% of the industry using wet milling and the 86% being dry grind [26]:

$$\left( \text{0.14} \right)\text{ 2.6}\frac{\text{gal}}{\text{bu}}\text{ + }\left( \text{0.86} \right)\text{ 2.785}\frac{\text{gal}}{\text{bu}}\text{ = 2.759}\frac{\text{gal}}{\text{bu}}$$

The DDGS yield for a conventional dry grind process was 17.56 lb (as is) per bushel or 15.69 lb (db) per bushel [26]. A dry matter content of 89.31% was assumed based on NRC data [51]. Oil contents in DDGS decreased from 10.43 to 8.90% db oil as a result of oil skimming [51], or

$$\frac{\text{10.43}\text{ }\text{-}\text{ }\text{8.90 lb oil}}{\text{100 lb DDGS}}\text{=1.53}\frac{\text{lb oil}\text{ }\text{recovered}}{\text{100 lb DDGS}}$$

Using the yield for DDGS (db) per bushel corn,

$$\text{15.69}\frac{\text{lb DDGS (db)}}{\text{bu corn}}\text{∙}\frac{\text{1.53 lb oil}}{\text{100 lb DDGS (db)}}\text{=}\text{ }\text{0.2401}\frac{\text{lb oil}}{\text{bu corn}}$$

Therefore, for ethanol plants that skim oil, the DDGS production rate is estimated to decrease to 17.56 – 0.2401 = 17.32 lb DDGS per bushel.

Assuming 50% of dry grind plants skim oil [26], the weighted dry grind yield of DDGS is:

$$\left( \text{0.50} \right)\frac{\text{15.69 lb db DDGS (no skim)}}{\text{bu }}\text{+}\left( \text{0.50} \right)\frac{\text{15.45 lb db DDGS (skim)}}{\text{bu }}\text{=}$$

$$\text{=}\text{15.57 }\frac{\text{lb DDGS}}{\text{bu }}\text{=17.43}\text{ }\frac{\text{lb DDGS}}{\text{bu }}\text{ at 89.31\% dry matter}$$

Similarly, the weighted oil recovery rate for dry grind plants is 0.1200 lb oil per bushel.

Using the current US ethanol production capacity of 14.71 B undenatured gal per year [27] and assuming a 50% adoption rate for skimming oil at a rate of 0.2401 lb oil per bushel, oil production is estimated as:

$$\left( \text{14.71 B gal} \right)\left( \text{0.86 dry grind capacity} \right)\left( \frac{\text{1 bu}}{\text{2.785 gal}} \right)$$

$$\left( \frac{\text{0.2401 lb oil}}{\text{bu}} \right)\left( \frac{\text{50}}{\text{100}}\text{plants skim} \right)\text{= 545}\text{.}\text{3 M lb oil skimmed}$$

This 545.3 M lb oil can be converted for use as biodiesel at a rate of 9 lb biodiesel per 10 lb oil. Since biodiesel has a density of 7.36 lb per gallon, the total biodiesel volume that can be produced from skimmed oil is:

$$\text{545.3 M lb oil}\left( \frac{\text{9 lb biodiesel}}{\text{10 lb oil}} \right)\left( \frac{\text{1 gal biodiesel}}{\text{7.36 lb biodiesel}} \right)\text{= 66.68 M gal biodiesel}$$

which is well below the U.S.-mandated biodiesel production of 1 B gal. It was assumed that biodiesel gallons from skimmed oil would replace gallons produced using soy oil. Because oil skimmed from the dry grind process is not economical for use in human food, this prevents using some of the soy oil for nonfood use.

*Scenario 5 (full starch) calculations*

The DDGS yield for a conventional dry grind process was 17.56 lb (as is) per bushel or 15.69 lb (db) per bushel [26]. Assuming 6% db residual starch content in DDGS from dry grind, the additional ethanol yield can be calculated:

$$\frac{\text{15.69 lb DDGS}}{\text{bu corn}}\left( \frac{\text{6 lb starch}}{\text{100 lb DDGS}} \right)\text{∙}$$

$$\text{ }\left( \frac{\text{1.11 lb glucose}}{\text{1 lb starch}} \right)\left( \frac{\text{0.5111 lb ethanol}}{\text{1 lb glucose}} \right)\left( \frac{\text{1 gal ethanol}}{\text{6.58 lb ethanol}} \right)\text{ = 0.081 }\frac{\text{gal}}{\text{bu}}$$

With this additional conversion of starch, the additional ethanol yield can be added to the current dry grind ethanol yield. Wet milling does not produce DDGS, so it was assumed that ethanol yields from wet milling are unchanged. Due to complete starch conversion during dry grind, the increased dry grind starch yield is

$$\text{2.785}\frac{\text{gal}}{\text{bu}}\text{ + 0.0812}\frac{\text{gal}}{\text{bu}}\text{ = 2.866}\frac{\text{gal}}{\text{bu}}$$

and the improved aggregate industry ethanol yield is:

$$\left( \text{0.14} \right)\text{ }\text{2.6}\frac{\text{gal}}{\text{bu}}\text{ + }\left( \text{0.86} \right)\text{ }\text{2.866}\frac{\text{gal}}{\text{bu}}\text{ = 2.829}\frac{\text{gal}}{\text{bu}}$$

The adjusted rate of DDGS production (no skimming) due to starch conversion is:

$$\text{15.69}\frac{\text{lb }\text{db }\text{DDGS}}{\text{bu}}\left( \text{1}-\frac{\text{6 lb starch removed}}{\text{100 lb DDGS}} \right)\text{ = 14.75 }\frac{\text{lb }\text{db }\text{DDGS}}{\text{bu corn}}$$

The adjusted rate of DDGS production (with skimming) due to starch conversion is:

$$\text{(15.69-}\text{0.2401}\text{)}\frac{\text{lb DDGS}}{\text{bu}}\left( \text{1}-\frac{\text{6 lb starch removed}}{\text{100 lb DDGS}} \right)\text{ = 14.52 }\frac{\text{lb DDGS}}{\text{bu corn}}\text{ (db)}$$

The weighted DDGS production rate (db) for 50% adoption of oil skimming by the dry grind industry is:

$$\left( \text{0.50} \right)\frac{\text{14.75 lb DDGS (no skim)}}{\text{bu }}+\left( \text{0.50} \right)\frac{\text{14.52 lb DDGS (skim)}}{\text{bu }}=\text{14.63 }\frac{\text{lb DDGS}}{\text{bu }}$$

Assuming 10.69% moisture (89.31% dry matter [51]), a 14.63 lb db per bushel DDGS yield is 16.38 lb (as is) per bushel DDGS.

NRC data provide DDGS compositions when skimming is performed commercially [51]. The DDGS protein contents are 27.3 and 27.4% without and with skimming, respectively; DDGS oil contents are 10.43 and 8.90% without and with skimming, respectively, then complete conversion of starch results in a protein content of:

$$\frac{\text{27.3 lb protein (no skim)}}{\text{100 lb DDGS}}\text{ }\frac{\text{100 lb DDGS (100\%)}}{\text{94 lb DDGS with full starch conversion}}\text{ = 29.04\% protein}$$

$$\frac{\text{27.4 lb protein (with skim)}}{\text{100 lb DDGS}}\text{ }\frac{\text{100 lb DDGS (100\%)}}{\text{94 lb DDGS with full starch conversion}}\text{ = 29.15\% protein}$$

And DDGS fat contents become

$$\frac{\text{10.43 lb oil (no skim)}}{\text{100 lb DDGS}} \frac{\text{100 lb DDGS (100\%)}}{\text{94 lb DDGS (full starch conversion)}}\text{ = 11.10\% oil}$$

$$\frac{\text{8.90 lb oil (with skim)}}{\text{100 lb DDGS}} \frac{\text{100 lb DDGS (100\%)}}{\text{94 lb DDGS (full starch conversion)}}\text{ = 9.468\% oil}$$

Assuming 50% adoption of oil skimming by the dry grind industry, the aggregate protein and oil compositions are:

$$\left( \text{0.50} \right)\text{29.04}\text{ }\text{+}\text{ }\left( \text{0.50} \right)\text{29.15}\text{ }\text{=}\text{ }\text{29.10\% protein }$$

$$\left( \text{0.50} \right)\text{11.10}\text{ }\text{+}\text{ }\left( \text{0.50} \right)\text{9.468}\text{ }\text{=}\text{ }\text{10.28\% oil }$$

*Scenario 6 (complete fiber) calculations*

Dien et al reported a 13.3% increase in ethanol yields when using a conventional yeast and a bacteria (E. coli FBR5) to convert C5 and C6 sugars [48]. The ethanol yield for dry grind would increase as 2.785(1+0.133) = 3.155 gal per bushel, or an increase of 2.785(0.133) = 0.3704 gal per bushel (2.437 lb ethanol per bushel). For this scenario, it is assumed that the wet milling sector would not adopt this technology, since it interferes with other processes at a wet mill. The weighted industry yield would increase to

$$\left( \text{0.14} \right)\text{ 2.6}\frac{\text{gal}}{\text{bu}}\text{ + }\left( \text{0.86} \right)\text{ 2.785}\text{(1+0.133)}\frac{\text{gal}}{\text{bu}}\text{ = }\text{3.078}\frac{\text{gal}}{\text{bu}}$$

The increased amount of conversion would result in more CO_2_ and less DDGS production as well as increased ethanol production. For the dry grind process which produces DDGS, the additional CO_2_ production from the additional conversion of starch and fiber is:

$$\text{(3.155}-\text{2.785)}\frac{\text{gal ethanol}}{\text{bu}}\left( \frac{\text{88 moles }\text{CO}_{\text{2}}}{\text{92 moles ethanol}} \right)\left( \frac{\text{6.58 lb ethanol}}{\text{1 gal ethanol}} \right)\text{=}\frac{\text{2.329 lb }\text{CO}_{\text{2}}}{\text{bu}}$$

To estimate the DDGS yield when converting residual starch and corn kernel fiber, the yield increases in ethanol and CO_2_ are subtracted from the current industry yield of 17.56 and 17.32 lb (as is) DDGS per bushel for no skimming and skimming, respectively (15.69 and 15.45 lb db per bushel, respectively). This gives

$$\text{17.56}\frac{\text{lb DDGS}}{\text{bu}}-\text{2.437}\frac{\text{lb} \text{ethanol}}{\text{bu}}-\text{2.329}\frac{\text{CO}_{\text{2}}}{\text{bu}}\text{ = }\text{12.794}\frac{\text{lb }\text{(as is) }\text{DDGS (no skim)}}{\text{bu}}$$

$$\text{17.32}\frac{\text{lb DDGS}}{\text{bu}}-\text{2.437}\frac{\text{lb} \text{ethanol}}{\text{bu}}-\text{2.329}\frac{\text{CO}_{\text{2}}}{\text{bu}}\text{= }\text{12.554}\frac{\text{lb }\text{(as is) }\text{DDGS (skim)}}{\text{bu}}$$

And a weighted DDGS yield, continuing to use 50/50 no skim/skim adoption, for the dry grind industry of

$$\left( \text{0.50} \right)\text{12.794}\frac{\text{lb DDGS (no skim)}}{\text{bu }}\text{+}\left( \text{0.50} \right)\text{12.554}\frac{\text{lb DDGS (skim)}}{\text{bu }}\text{=}\text{12.674}\frac{\text{lb }\text{(as is) }\text{DDGS}}{\text{bu }}$$

A 13.3% increase in ethanol yield originates from carbohydrates (starch and fiber) that otherwise would have been present in the DDGS. The 2.437 lb ethanol per bushel was converted from carbohydrate that would have become DDGS, so the amount of carbohydrate converted can

be calculated as:

$$\left( \frac{\text{2.437 lb ethanol}}{\text{bu}} \right)\left( \frac{\text{1 lb glucose}}{\text{0.5111 lb ethanol}} \right)\left( \frac{\text{1 lb carbohydrate}}{\text{1.11 lb glucose}} \right)\text{=}\frac{\text{4.296 lb carbohydrate}}{\text{bu}}$$

In other words, 4.296 lb per bushel of carbohydrate material originally recovered as DDGS was converted to ethanol and CO_2_. As a result, this 4.296 lb carbohydrate per bushel is removed from the DDGS and affects protein and oil contents. The new DDGS composition can be calculated from NRC data [51] for conventional dry grind plants and those that do skimming:

$$\frac{\text{27.3 lb protein (no skim)}}{\text{100 lb DDGS}}\text{ }\frac{\text{100 lb DDGS (100\%)}}{\text{(100}-\text{4.296) lb DDGS with starch and fiber conversion}}\text{ }$$

$$\text{= 28.525\% protein}\text{ 'as is'}\text{ (no skim)}$$

$$\frac{\text{27.4 lb protein (with skim)}}{\text{100 lb DDGS}}\text{ }\frac{\text{100 lb DDGS (100\%)}}{\text{(100}-\text{4.296) lb DDGS with starch and fiber conversion}}\text{ }$$

$$\text{= 28.630\% protein}\text{, as is }\text{(skim)}$$

$$\frac{\text{10.43 lb oil (no skim)}}{\text{100 lb DDGS}}\text{ }\frac{\text{100 lb DDGS (100\%)}}{\text{(100}-\text{4.296) lb DDGS with starch and fiber conversion}}\text{ }$$

$$\text{= 10.898\% oil}\text{, as is}\text{ (no skim)}$$

$$\frac{\text{8.90 lb oil (with skim)}}{\text{100 lb DDGS}}\text{ }\frac{\text{100 lb DDGS (100\%)}}{\text{(100}-\text{4.296) lb DDGS with starch and fiber conversion}}\text{ }$$

$$\text{= 9.300\% oil}\text{, as is}\text{ (skim)}$$

With a 50% adoption rate of oil skimming for the industry, the aggregate compositions are

(0.50)(28.525) + (0.50)(28.630) = 28.58% protein

(0.50)(10.898) + (0.50)(9.300) = 10.10% oil

* Full citations for references given in main paper; abbreviations documented there also.
